# Effect of Temperatures and Moisture Content on the Fracture Properties of Engineered Cementitious Composites (ECC)

**DOI:** 10.3390/ma15072604

**Published:** 2022-04-01

**Authors:** Shuling Gao, Puxu Xie

**Affiliations:** 1 School of Civil and Transportation Engineering, Hebei University of Technology, Tianjin 300401, China; xpx3136628063@163.com; 2 Civil Engineering Technology Research Center of Hebei Province, Tianjin 300401, China

**Keywords:** ECC, low temperatures, saturation, freeze–thaw, fracture toughness, fracture energy

## Abstract

This research will help to improve our understanding of the fracture properties of ECC at low temperatures (long-term low temperatures, freeze–thaw) and evaluate the safety properties of ECC under low-temperature conditions. Three levels of saturation (saturated, semi-saturated, and dry), four target temperatures (20, 0, −20, and −60 °C), and the effect of the coupled of the two on the mode I fracture properties of ECC were investigated. Then, we compared and analyzed the fracture properties of ECC loaded at 20 and −20 °C, after different freeze–thaw cycles (25, 50, 100 cycles), which were compared with saturated specimens without freeze–thaw at the four target temperatures to analyze the differences in low-temperature and freeze–thaw failure mechanisms. Temperatures and saturation have a significant effect on the fracture properties. Low temperatures and freeze–thaw treatments both decreased the nominal fracture energy of ECC. Distinct differences in matrix and fiber-matrix interface damage mechanisms have been discovered. Low temperatures treatment transforms ECC from a ductile to a brittle fracture mode. However, even after 100 freeze–thaw cycles, it remains ductile fractured. This study complements the deficiencies of ECC in low-temperature theoretical and experimental applications, and it sets the stage for a broad range of ECC applications.

## 1. Introduction

ECC (engineered cementitious composite) is a special high-performance, fiber-reinforced, cement-based composite, which exhibits excellent strain-hardening behavior during overstretching the loading process [1,2,3,4]. It is equipped with several excellent properties, including high ductility, superior crack control, self-healing, and environmental protection. The design theory of ECC is based on micromechanics and fracture mechanics, and the stress concentration, caused by external load, is relieved by multi-crack mode [5]. Work with cracks is its common state. While the traditional strength theory of ECC reflects the mechanical properties of ECC as a whole, it ignores the non-homogeneity of ECC materials and, thus, cannot fully describe the ECC process from the crack initiation, development, connection, and formation of macroscopic cracks to component failure. Moreover, in practical engineering applications, ECC may generate cracks for a variety of reasons (such as drying shrinkage, temperatures stress, external load, foundation deformation, etc.), and serious cracks can jeopardize the integrity and stability of the structure, as well as have a significant impact on the safety of the structure. Nowadays, in actual engineering structures, cracks in the material always occur, posing possible safety issues, due to the “fracture” of the structure. In terms of structural safety assessment and crack analysis, fracture mechanics are used to investigate the fracture properties of fiber cement-based reinforced materials [6], which has garnered the attention and approval of many scholars.

ECC has a wide range of potential applications and offers significant economic benefits. Following the Three Gorges Project, the promotion of the South-to-North Water Transfer Project, and the strategy for large-scale development in western China has resulted in the extensive construction of a variety of large-scale water conservation and transportation projects, including the foundations for river-crossing bridges [6], piers, dams, etc. Considering the excellent properties of ECC, it is expected to be applied to these constructions [7,8]. The water level of these wading structures, on the other hand, often changes, and ECC has varying water saturation levels in practical applications. Additionally, winter temperatures in cold places are far too low, and low temperatures have historically been regarded as one of the extreme conditions for the application of concrete. Some countries, including Sweden, experience a rainy autumn before winter; if ECC is applied in this environment, it is affected by the water and low temperatures, and the structure of wading constructions at low temperatures will face the coupling condition of low temperatures and varying water saturation levels.

Zhang P et al. (2010) discovered that the moisture content of concrete impacts its fracture properties, with entirely dry concrete having fracture energy 1.18 times that of concrete with 75% water saturation level and 1.57 times that of concrete with 100% water saturation level [9]. Additionally, they observed that the deformability of concrete gradually weakens as the water saturation level increases, which is consistent with the findings by Guohui Z (2016) that water intrusion into concrete is detrimental to its crack resistance, and saturated concrete “becomes brittle” [10]. According to Planas J (1989) [11] and Maturana P (1990) [12], the higher the water saturation level of the concrete specimens, the greater the increase in fracture energy as temperatures decrease. They believe that the increase in fracture energy is primarily caused by water freezing at low temperatures. Fan (2020) used the double-K fracture criterion to investigate the fracture properties of concrete specimens at various temperatures (20, 0, −20, and −40 °C) and discovered that both initial fracture toughness and unstable fracture toughness increase with decreasing temperatures [13]. Weimin Q (2021) studied the fracture properties of UHTCC (ultra-high toughness cementitious composite) with varying fiber contents at low temperatures (20, 0, −40, −80, −120, and −160 °C) and found that the initial fracture load and peak load of UHTCC increase as temperatures decrease [14]; UHTCC is another name for ECC. Concrete’s tensile and compressive properties are significantly affected by water saturation and low temperatures [15,16]. According to Zhengwu J (2018), this is due to the filling effect of pore ice at low temperatures, which “repairs” the cement-based materials’ poor pore structure and further increases their tensile and compressive properties [17]. ECC is a type of material that has high ductility and energy consumption, due to fiber sliding hardening [2]. It is unknown how fracture properties change with varying water saturation levels and the coupling of low temperature and varying water saturation levels; hence, its safety performance at low temperatures cannot be determined.

Additionally, some areas are not permanently in a low-temperature environment. Yet, wading constructions with a variable climate and a high temperatures difference between day and night are susceptible to freeze–thaw damage. Internal tensile stress is caused by frozen water during freeze–thaw cycles [18], which manifests as irreversible tensile strain and microcracks [19,20]. Therefore, freeze–thaw damage can be viewed as a complex fracture propagation process [21]. According to Dong Y (2018), microcracks, caused by freeze–thaw cycles, are the primary reason for the degradation of mechanical properties of concrete [22]. Goszczyńska (2012) reported that fracture parameters are sensitive to the microstructural changes caused by the accumulation of damage within the material, as a result of repetitive actions [23]. According to Zhiqiang H et al. (2018), the initial fracture toughness, unstable toughness, and fracture energy of ECC specimens decrease as the number of freeze–thaw cycles increases [24]. They discovered that the decline in unstable fracture toughness is greater than the decline in initial fracture toughness. The aforementioned studies do not consider the circumstance in which ECC is subjected to low temperature (−20 °C) and freeze–thaw coupled damage. Low temperatures, combined with freeze–thaw, have a greater damaging effect on the structure.

To investigate the fracture properties of ECC, under a variety of low-temperatures conditions, the three-point bending method recommended by DL/T5332-2005 “Norm for Fracture Test of Hydraulic Concrete” was used. To investigate the effect of three typical water saturation levels (saturated, semi-saturated, and dry), four target temperatures (20, 0, −20, and −60 °C), and their interaction on the fracture properties of ECC in the low-temperature environment were compared and analyzed, as well as the difference in fracture properties loaded at 20 and −20 °C after different freeze–thaw cycles and fracture failure mechanism of ECC with initial fracture defects at low temperatures and freeze–thaw cycles.

## 2. Materials and Methods

### 2.1. Material and Sample Preparation

Beijing Sansui Wenyuan New Building Materials Co., Ltd.(Beijing, China). East Hebei ordinary Portland cement (P·O 42.5 grade, OPC), Hebei Jiegui Mineral Products Co., Ltd. (Hebei, China). Fly ash (grade I, FA), Tianjin Yandong Haotian Mineral Products Co., Ltd. (Tianjin, China) Silica sand (70–140 meshes), Gansu Sanyuan Silicon Material Co., Ltd. (Gansu, China) Silica fume (SF) with SiO_2_ content up to 94%, Sika water reducer 540p powdered polycarboxylate acid superplasticizer (Shanghai Kaiyin Chemical Co., Ltd., Shanghai, China) and K-II REC15 PVA fiber (Kuraray, Shanghai, China) were used. Table 1 shows the chemical composition of fly ash and silica fume. Parameters of PVA fiber are given in Table 2. The mix proportion of ECC are listed in Table 3.

**Figure 1:**
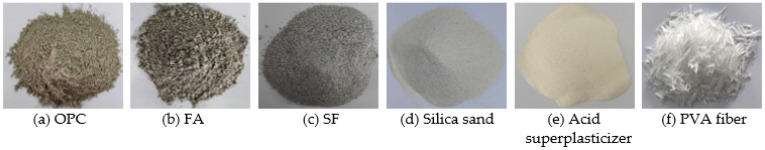
Experimental materials.

The composition of the materials are shown in Figure 1.

Select the three-point bending test specimen according to the literature [25,26]. The height of the ECC specimen was selected as 100 mm; at the same time, to ensure that the S/h = 4, the effective span (S) was selected as 400 mm, d is 20 mm to ensure a good overlap between the specimen and the support. The size of 440 × 100 × 100 mm three-point bending beam (TPB) specimens was produced, and all specimens were demolded 24 h after pouring, according to GB/T 50081-2002, under standard curing conditions (20 °C, 95% relative humidity) for 28 d [27].

**Figure 2:**
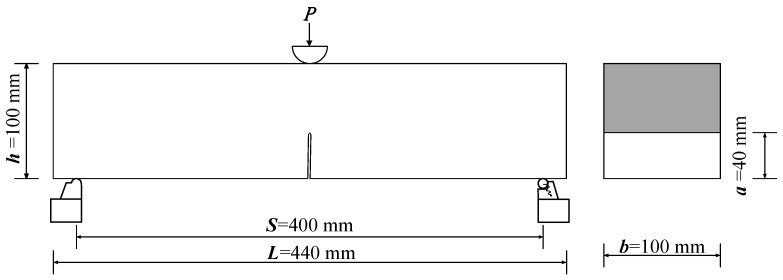
The specific dimensions of the test specimen section. Note: a is the depth of the notch (40 mm), b is the thickness of the test specimen (100 mm), S is the net span of the test specimen (400 mm), L is the length of the test specimen (440 mm), h is the height of the test specimen (100 mm), P is the applied load (kN).

The specific dimensions of the test specimen are shown in Figure 2.

The ECC low temperatures fracture experiment consists of two parts. The first part uses four target temperatures T= (20, 0, −20, and −60 °C). Each group of samples is subjected to a three-point bending fracture test at the same temperature target, with three levels of saturation (saturated, semi-saturated, and dry).

The second part is the three-point bending fracture test at different temperatures (20 and −20 °C), following different freeze–thaw cycles (25, 50, and 100), and the mass-loss rate and dynamics are measured every 25 cycles.

Their serial numbers are provided in Table 4.

### 2.2. Water Saturation Control

The following methods are used to make saturated, semi-saturated, and dry specimens [10,28].

A saturated specimen is prepared after curing the specimen under standard curing conditions (20 ± 2 °C, relative humidity 95%) for 28 days: the specimen is placed in a water tank, tap water is added to level the water surface level with the top surface of the specimen. The test specimen should still be submerged after absorbing water. When weighing, the surface should be wiped with a damp cloth, so to remove any clear water but still keep it in a wet state. After the first 12 h, the test is repeated every hour, every other day. The test is repeated until the quality does not change for three consecutive days, at which point it is considered saturated.

A dry specimen is prepared after curing the test specimen for 28 days under standard curing conditions (20 ± 2 °C, relative humidity 95%). This is achieved by weighing the mass of the test specimen after soaking and recording its saturated water quality, placing it in an electric heating blast drying oven with constant temperatures, and baking at 60 °C, until the vaporized mass of the surface area per unit of time is less than 0.002 kg/(h·m^2^); thus, it is deemed to have reached a completely dry state.

A semi-saturated specimen is prepared after the specimens have been cured for 28 days under standard curing conditions (20 ± 2 °C, relative humidity 95%). According to Formula (1), the moisture content is calculated, and the dry specimen is placed into a water tank and weighed every half an hour. When the water absorption reaches half of the value calculated using the Formula (1) in the saturated state, it is removed from the water tank and regarded as a semi-water-retaining state.

(1) $$\begin{array}{c} W_{c}=\frac{M_{1}-{\;M}_{0}}{M_{0}} \end{array}$$

where $W_{c}$ is the moisture content of TPB, $M_{1}$ is the mass of the TPB after soaking in water (g), and $M_{0}$ is the initial TPB mass (g).

In practical engineering applications, the structure may be in different saturated states. At this time, non-destructive testing moisture content testing can be used to measure the actual moisture content of the structure [29,30,31]. An approximate evaluation of fracture properties at low-temperature conditions with different moisture content forms the basis of this study. The dried specimen is absorbed to a saturated state after the test, according to the water saturation control method described in this article, and the moisture content change is shown in Figure 2.

### 2.3. Freeze–Thaw Cycles Test Method

The model JB-TDRF-28F concrete rapid freeze–thaw cycle test machine, produced by Shanghai Jiaben Test Equipment Co., Ltd. (Shanghai, China), was used. According to the quick-freezing method in GB/T50082-2009 “Standard for Long-term Performance and Durability Test Methods of Ordinary Concrete”, the microcomputer’s automatic concrete rapid freeze–thaw test equipment is used [32]. After curing for 28 days, the specimen is immersed for five days to make it in a saturated state, measure the initial mass $W_{0}$, and transverse fundamental frequency $f_{0i}$ after immersing the specimen. We measured its transverse fundamental frequency and quality every 25 cycles, and fracture properties tests were performed at 20 and −20 °C at 25, 50, and 100 freeze–thaw cycles.

(2) $$\begin{array}{c} \;\;\;\;\;\;\;\;\;\;\;\;\;\;\;\;\;\;\;\;\;\;\;\;\;\;\;\;\;\;\;\;\;\;\;\;\;\;\;\;\;\;\;\;\;\;\;\;\;\;\;\;\;\;\;\;\;\;\;\;\;\;\;\;\;P_{\mathrm{ni}}=\frac{f_{\mathrm{ni}}^{2}}{f_{0i}^{2}}\;\times \;100\% \end{array}$$

(3) $$\begin{array}{c} \;\;\;\;\;\;\;\;\;\;\;\;\;\;\;\;\;\;\;\;\;\;\;\;\;\;\;\;\;\;\;\;\;\;\;\;\;\;\;\;\;\;\;\;\;\;\;\;\;\;\;\;\;\;\;\;\;\;\;\;\;\;P_{i}=\frac{1}{3}\overset{3}{\underset{n=1}{\sum }}P_{\mathrm{ni}} \end{array}$$

where $P_{\mathrm{ni}}$ is the relative dynamic elastic modulus of the *i*-th specimen after n freeze–thaw cycles (%), $f_{\mathrm{ni}}$ is the transverse fundamental frequency of the i-th specimen after n freeze–thaw cycles (HZ), $f_{0i}$ is the initial value of the transverse fundamental frequency of the *i*-th specimen before the freeze–thaw cycles (HZ), and $P_{i}$ is the average value of the three measurements of the dynamic elastic modulus of the specimen after freeze–thaw cycles (%).

**Figure 3:**
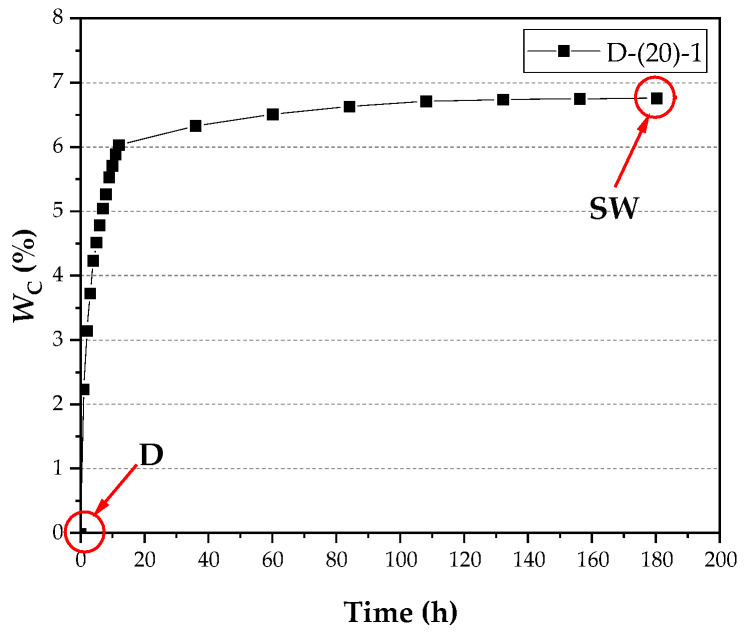
Variation of moisture content with immersion time.

The test results of its mass-loss rate and relative dynamic elastic modulus are shown in Figure 3. The mass loss rate calculation formula is shown below (4):

(4) $$\begin{array}{c} \Delta W_{n}=\frac{W_{n}-W_{0}}{W_{0}} \end{array}$$

where $\Delta W_{n}$ is the mass loss rate of the specimen after n freeze–thaw cycles (%), $W_{0}$ is the mass of the specimen before freeze–thaw (g), and $W_{n}$ is the mass of the specimen after *n* freeze–thaw cycles (g).

**Figure 4:**
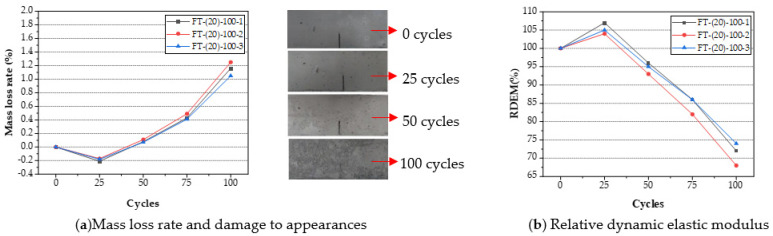
Changes in mass-loss rate and damage to appearances (**a**) and relative dynamic elastic modulus after n times of freeze–thaw cycles (**b**).

The mass loss rate, appearance, and relative dynamic elastic modulus after freeze-thaw cycles are shown in Figure 4.

### 2.4. Test Setup and Instrumentation

#### 2.4.1. 20 °C Mode I Fracture Test Setup

**Figure 5:**
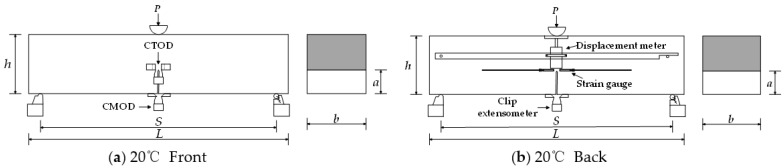
Measuring points of three-point beading test at 20 °C. (**a**) 20 °C Front. (**b**)−20 °C Back.

Measuring points of the three-point beading test at 20 °C are shown in Figure 5.

Two strain gauges were symmetrically pasted at the left and right sides of the front of the initial notch, and each strain gauge had a horizontal distance 5 mm away from the crack tip. Each measuring point data was recorded by a half-bridge, consisting of a working and compensation strain gauge. The strain changes of ECC in fracture tip areas were detected to determine the cracking load [33].

The clip-on extensometer, with a range of ±10 mm, records the crack mouth opening displacement (CMOD), and the clip-on extensometer with a range of ±5 mm records the crack tip opening displacement (CTOD).

#### 2.4.2. The 0, −20, and −60 °C Mode I Fracture Test Setup

**Figure 6:**
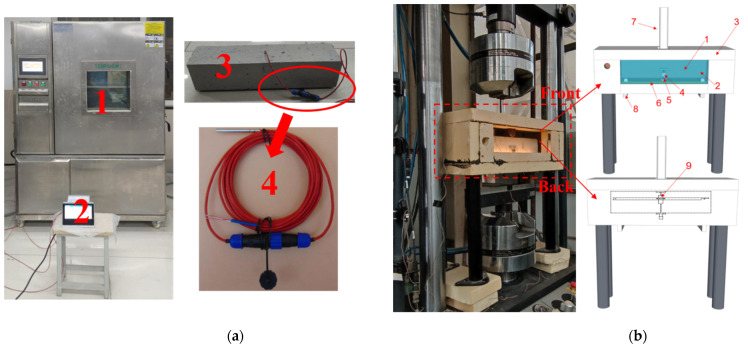
Cooling system and test setup (0, −20, and −60 °C). (**a**) Cooling system. Note: 1—cooling refrigerator; 2—temperatures recorder; 3—temperatures control test specimen; 4—temperatures sensor. (**b**) Test setup. Note: 1—ECC specimen; 2—acrylic glass (observation window); 3—clip extensometer (CTOD) 4—clip extensometer (CMOD); 5—polycyanurate (heat preservation); 6—Led light (illumination); 7—loading head; 8—distribution beam; 9—displacement meter (using non-thermal conductive resin to make the clamping end).

The temperature dropping specimen and temperature control specimen of the internally-embedded temperature sensor were made of the same size and material as the loading specimen. Then, the temperature control, temperature dropping, and loading specimens were cooled in the refrigerator, with an operating temperature of 20~−80 °C, developed by Suzhou Jiang Kai Machinery Equipment Co., Ltd. (Suzhou, China). A temperature control recorder was used to ensure that the central temperature of the temperature control specimen dropped to the experimental temperature. After that, the temperature dropping specimen was moved into the self-made insulation can to precool the loading environment and minimize the temperature loss during the experiment. As the temperature in the insulation can was lowered, the loading specimen was moved into the insulation can to be loaded. Cooling system and low temperature test setup (0, −20, and −60 °C) are shown in Figure 6.

**Figure 7:**
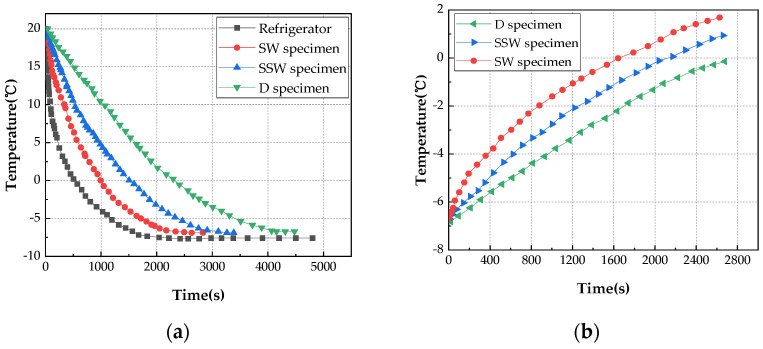

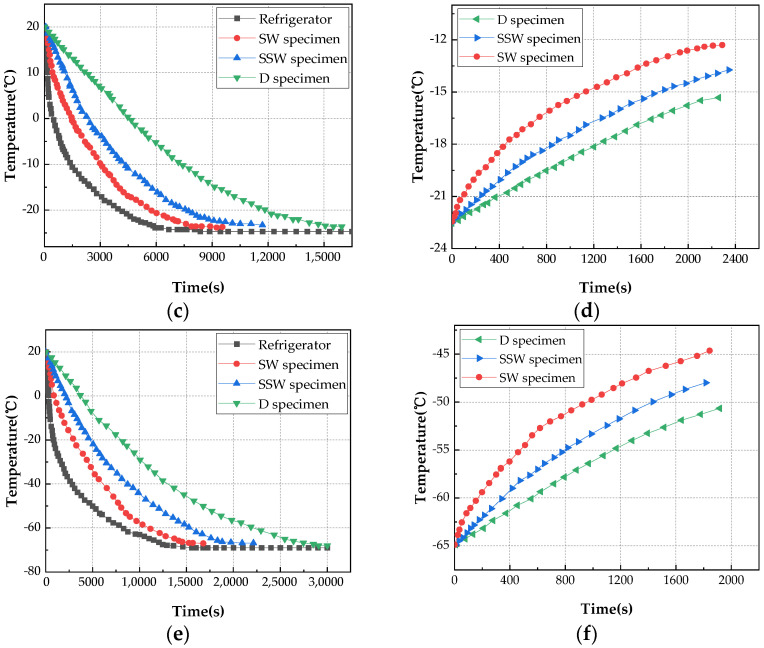
Heating and cooling curves of SW, SWW, and D specimens at 0, −20, and −60 °C; (**a**) 0 °C cooling curve; (**b**) 0 °C heating curve; (**c**) −20 °C cooling curve; (**d**) −20 °C heating curve; (**e**) −60 °C cooling curve; (**f**) −60 °C heating curve.

Since the constant temperature cannot be maintained during the test, in order to be as close to the target temperature as possible during the loading process, the temperature of the specimen is lower than the target temperature during initial loading. The heating and cooling curves of the specimen are shown in Figure 7. The heating and cooling curves are from the test results of temperature-controlled specimens with different saturation levels. All specimens were loaded within 30 min.

## 3. Results Analysis and Discussion

### 3.1. ECC Fracture Properties with Different Saturation in Low-Temperatures Environment

To judge the ECC fracture properties, Liu Wen (2012) pointed out that, for the ECC fracture areas, the opening displacement of the crack opening is more commonly used than the mid-span deflection, using double J integral ($J_{IC}$ fracture energy and ($J_{\mathrm{IF}}$ failure fracture energy) and ductile fracture index $I_{D}$, J-R resistance curve, and redefining ΔA can avoid the tedious work of flexibility calibration for J [34]. However, the aim is to obtain accurate results, based on the J-R resistance curve; one needs to divide a very dense grid. The frost and fog on the specimen’s surface will form at low temperatures, blocking the cracks and original grid lines. Some cracks in the specimen will be repaired after unloading. Counting the number of grids penetrated by the fracture precisely is challenging, whether during or after loading. ECC has high toughness, multi-slit cracking, and the material undergoes ductile fracture. There is more than one fracture surface. Therefore, the linear elastic fracture mechanics theory cannot be directly used to assess the stability resistance of the composite materials. The capacity and instability toughness calculation is not the true instability toughness. However, the main crack still dominates in fracture propagation, and it can approximate the ability of strain-hardening composites to resist instability, which is referred to as nominal instability toughness here.

According to DL/T5332-2005, hydraulic concrete fracture test regulations, the calculation formula of initiation toughness and unstable toughness are as follows [35].

(5) $${\;K}_{IC}^{ini}=\frac{3\left(P_{ini}+\frac{mg}{2}{\;\times \;10}^{-2}\right){\times 10}^{-3}\;\times \;S\;\times \;\sqrt{a}}{{2bh}^{2}}f(\alpha )$$

(6) $$\begin{array}{c} \;\;\;\;\;\;\;\;\;\;\;\;\;\;\;\;\;\;\;\;\;\;\;\;\;\;\;\;\;\;\;\;\;\;\;\;\;\;\;K_{IC}^{un}=\frac{3\left(P_{max}+\frac{mg}{2}{\;\times \;10}^{-2}\right){\;\times \;10}^{-3}\;\times \;S\;\times \;\sqrt{a}}{{2bh}^{2}}{f(\alpha }_{c}) \end{array}$$

where *m* is the mass of the specimen (mg), *g* is the acceleration due to gravity, *g* = 9.81 m/s^2^, *h* is the height of the specimen (mm), *S* is the net span of the beam (mm), *b* is the thickness specimen (mm), and *a* is the initial crack length of the specimen (mm).

f(α) and ${f(\alpha }_{c})$ are geometrical factors [36], which can be obtained by Formulas (7) and (8).

(7) $$\begin{array}{c} \;f\left(\alpha \right)=\frac{1.99\;-\;\alpha (1\;-\;\alpha )[2.15\;-\;3.93\alpha +2{.7\alpha }^{2}]}{(1+2\alpha )(1\;-{\;\alpha )}^{3/2}} \end{array}$$

(8) $$\begin{array}{c} \;f\left(\alpha_{c}\right)=\frac{1.99\;-\;\alpha (1\;-\alpha_{c})[2.15\;-\;3{.93\alpha }_{c}+2{.7\alpha }_{c}^{2}]}{{(1+2\alpha }_{c})(1\;-{\;\alpha }_{c}{)}^{3/2}} \end{array}$$

where ${\alpha =(a+h}_{0}{)/(h+h}_{0})$, $\alpha_{c}{=(a+h}_{0}{)/(h+h}_{0})$, $h_{0}$ is the thickness of the blade used to fix the clip, and $\alpha_{c}$ is the effective elastic crack length corresponding to the peak load (9).

(9) $$\begin{array}{c} \;\;\;\;\;\;\;\;\;\;\;\;\;\;\;\;\;\;\;\;\;\;\;\;\;\;\;\;\;\;\;\;\;\;\;\;\;\;\;\;\;\;\;\;\;\;\;\;\;\;\;\;\;\;\;\;\;\;\;\;\;\;\;\;\;a_{c}=\frac{2}{\pi }\left({h+h}_{0}\right)\arctan{\left(\frac{E\cdot b\cdot {CMOD}_{c}}{32{.6P}_{max}}-\;0.1135\right)}^{\frac{1}{2}}-{\;h}_{0} \end{array}$$

where ${CMOD}_{c}$ value corresponds to the peak load.

The elastic modulus, E, can be obtained by simplified linear elastic fracture formulas (10) and represents the initial compliance coefficient, which is calculated from CMOD and P at any point on the linear ascending stage of the P-CMOD curve (11).

(10) $$\begin{array}{c} \;E=\frac{1}{{bc}_{i}}[3.70+32{.60\tan}^{2}(\frac{\pi }{2}\cdot \frac{{a+h}_{0}}{{h+h}_{0}})] \end{array}$$

(11) $$\begin{array}{c} \;\;\;\;\;\;\;\;\;\;\;\;\;\;\;\;\;\;\;\;\;\;\;\;\;\;\;\;\;\;\;\;\;\;\;\;\;\;\;\;\;\;\;\;\;\;\;\;\;\;\;\;\;\;\;\;\;\;\;\;\;\;c_{i}=\frac{{CMOD}_{i}}{P_{i}} \end{array}$$

According to the Japanese JCI-S-001-2003 standard, the formula for calculating the nominal fracture energy of the TPB specimen, which can be obtained by (12) and (13) [37].

(12) $$\begin{array}{c} \;\;G_{I}=\frac{0{.75W}_{0}{+W}_{1}}{A_{tig}} \end{array}$$

(13) $$\begin{array}{c} \;\;\;W_{1}=0.75(\frac{S}{L}m_{1}{+2m}_{2})g\cdot {CMOD}_{0} \end{array}$$

where $W_{0}$ is the area under the *P-CMOD* curve, $A_{tig}$ is the area of the broken ligament, *S* is the loading span, *L* is the total length of the specimen, $m_{1}$ is the mass of specimen, $m_{2}$ is the weight of the loading head not fixed on the testing machine $m_{2}$ = 0 kg, *g* is the gravity acceleration, and ${CMOD}_{0}$ is the corresponding *CMOD* value, when ${CMOD}_{0}$ is taken as the corresponding *CMOD* value and the bearing capacity drops to 20% of the peak load.

The fracture energy is the same as the unstable fracture toughness, which is an approximate evaluation of the ECC fracture properties; here, it is called the nominal fracture energy [38].

The calculation of fracture energy, by *P-CMOD* curve, can avoid the size effect and reduce the influence caused by the plastic deformation of the support [39]. Compared with the calculation of fracture energy by the P−δ curve, it has more advantages. Hence, the *P-CMOD* curve is used to calculate fracture energy. P−δ and *P-CMOD* are basically linear; in order to avoid repetition, only *P-CMOD* curves are given [39]. If the difference between the average, maximum, and the minimum of the peak load value was less than 15%, then take the average value of the three specimens as the test value; otherwise, the closest to the average value is selected as the test value.

**Figure 8:**
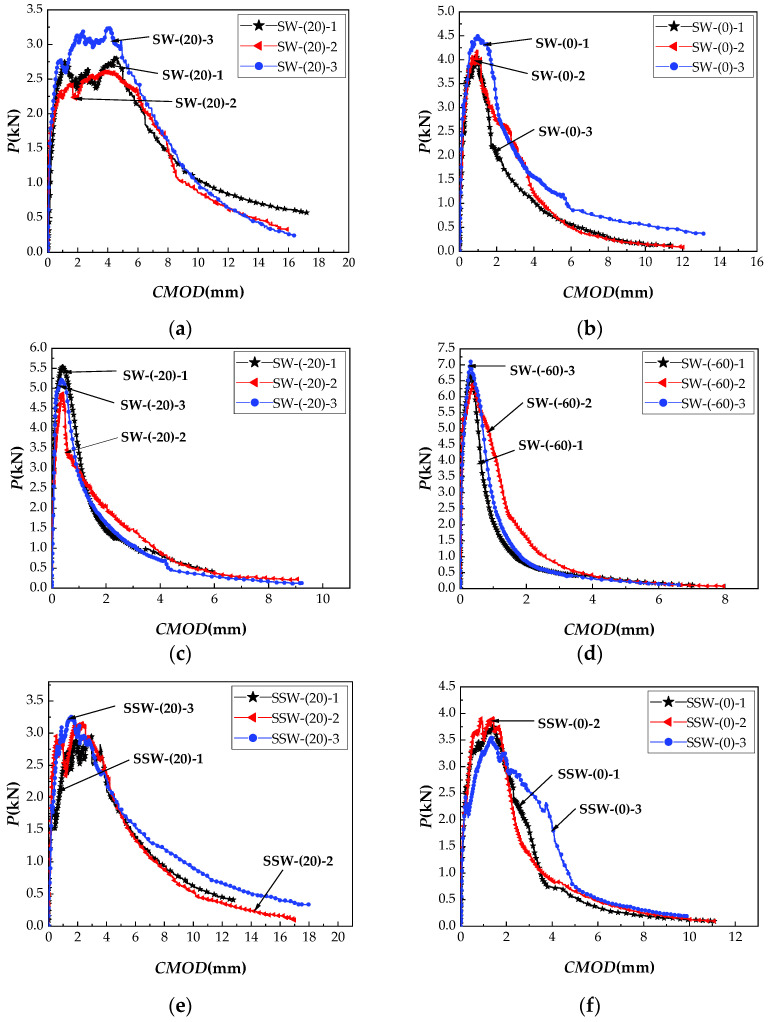

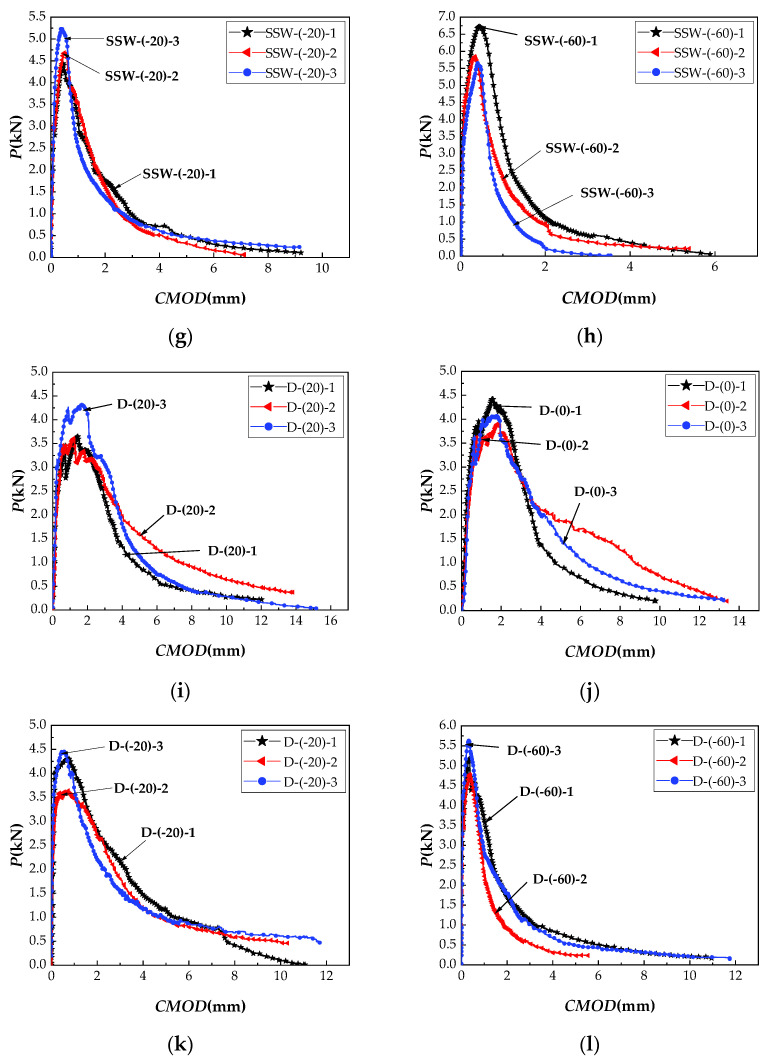
ECC *P-CMOD* curves of three levels of saturation in four target temperatures. (**a**) SW group at 20 °C; (**b**) SW group at 0 °C; (**c**) SW group at −20 °C; (**d**) SW group at −60 °C; (**e**) SSW group at 20 °C; (**f**) SSW group at 0 °C; (**g**) SSW group at −20 °C; (**h**) SSW group at −60 °C; (**i**) D group at 20 °C; (**j**) D group at 0 °C; (**k**) D group at −20 °C; (**l**) D group at −60 °C.

The *P-CMOD* curves of SW, SSW, and D at different temperatures (20, 0, −20, and −60 °C) specimens are shown in Figure 8a–l, and the calculated results of fracture properties are listed in Table 5.

**Figure 9:**
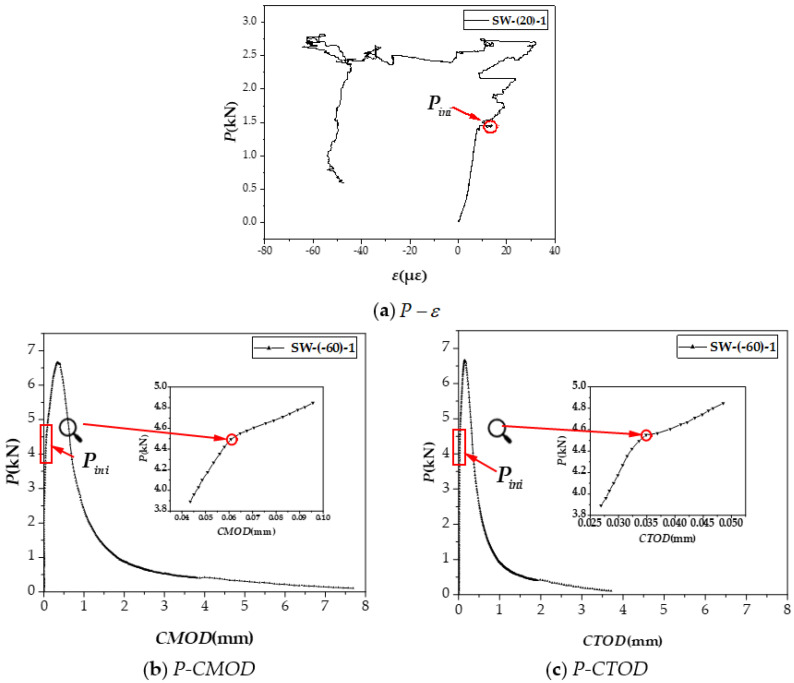
The methods of determining the crack initiation forces at four target temperatures. (**a**) P−ε. (**b**) *P-CMOD.* (**c**) *P-CTOD*.

The methods of determining the crack initiation forces at four target temperatures are shown in Figure 9.

Figure 9a shows that, before the ECC cracks, the load-strain (P−ε) relationship is linear. When the ECC cracks, the strain energy is released, and the first strain retraction point appears on the P−ε curve. Since ECC exhibits multi-slit cracking, the bridging effect of fibers allows the strain near the tip of the notch to continue to increase when the load increases, after retraction, occur [40]. Therefore, the first strain turning point on the P−ε curve is defined as $P_{ini}$ of ECC. At low temperatures, strain gauges are no longer useful; hence, the load corresponding to the turning point from the linear to the nonlinear segments of [41] *P-CMOD* and *P-CTOD* are used as P−ε, and the methods are displayed in Figure 9b,c.

#### 3.1.1. The Influence of Three Levels Saturation on the Fracture Properties of ECC at Various Target Temperatures

To compare and study the deformation capacity in the ECC *P-CMOD* curve, with varied saturation at the same temperatures, the test specimen closest to the average value of the *P-CMOD* curve is selected. This data selection method is based on the literature [42].

**Figure 10:**
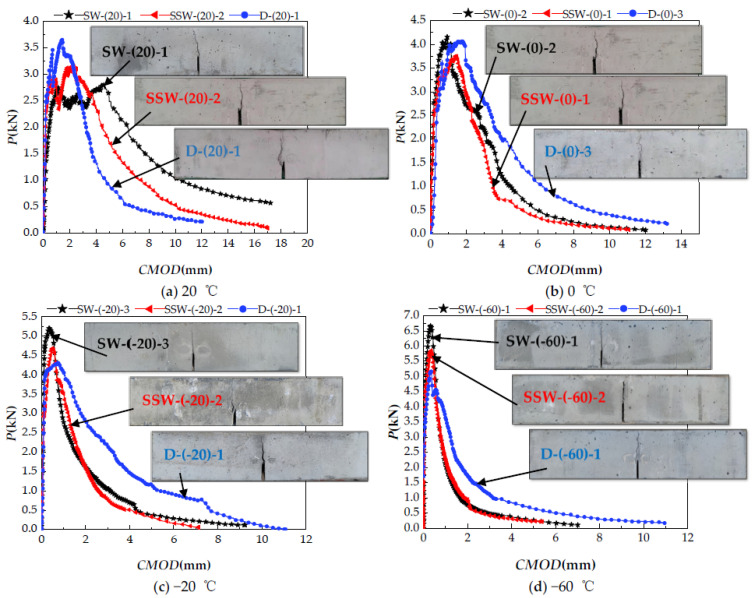
*P-CMOD* curve and fracture morphology of SW, SSW, and Dat; (**a**) 20 °C; (**b**) 0 °C; (**c**) −20 °C; (**d**) −60 °C.

The *CMOD* curve of the specimen that is close to the average value is shown in Figure 10a–d. It should be noted that, because this experimental study is for the same size specimens under different working conditions, the double *K* criterion is used for calculation and, at this time, $P_{ini}$ and $K_{IC}^{ini}$ are positively correlated. Compared and analyzed the changes of $K_{IC}^{ini}$, $K_{IC}^{un}$, and $G_{I}$, the results are shown in Figure 10a–d. During the test, the inside of the incubator could not maintain a constant temperature, resulting in frost and fog on the surface of the test specimen and observation window, and it was impossible to observe the changes in the crack growth pattern during the loading process; hence, only the fracture morphology was given.

Figure 10 depicts the failure morphology of the specimens at various saturation and temperatures. The lower the temperatures, the narrower the angle of the main cracks when the specimens are destroyed and fewer the number of cracks around the prefabricated cuts of the specimens.

**Figure 11:**
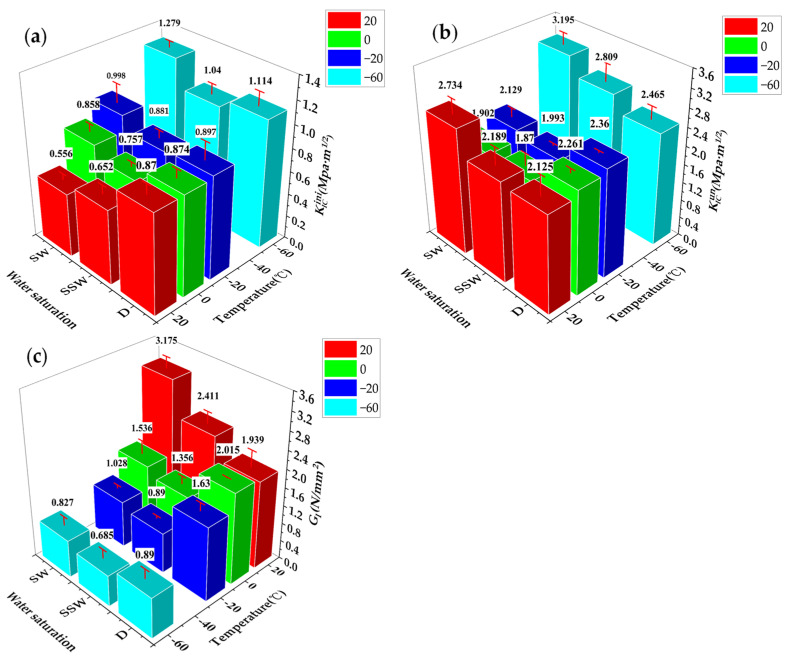
Changes in (**a**) $K_{IC}^{ini}$, (**b**) $K_{IC}^{un}$, and (**c**) $G_{I}$ at different saturation levels at the same target temperature.

Figure 11 shows that, at 20 °C, no matter the water saturation level, all of the failures are ductile, with obvious strain hardening, $K_{IC}^{ini}$ D > SSW > SW. This can be explained from the energy perspective. External forces must overcome the surface energy of microcracks formation and calcium silicate hydrate during the generation, convergence, and propagation of cracks in ECC [43]. The main cement hydration product, CSH gel, determines the cohesive force of cement paste. The mechanical properties of cement-based materials are determined by the interaction between water and CSH gel. The penetration of water molecules causes the CSH gel to change from an amorphous structure to a layered structure as the saturation rate increases. Therefore, when water infiltrates the ECC, it reduces the van der Waals force between the microscopic particles of the material, weakening the cohesion between the particles on the ECC surface, thereby reducing the surface energy, as well as the energy required to form a new fracture surface. The bridge bond between calcium silicate tablets varies from a simple ${Ca}_{w}-O_{s}$ bond to a combination of ${Ca}_{w}-O_{s}$ and H− bonds, as well as ${Ca}_{w}-O_{s}$ with a higher potential energy. Furthermore, in the dry specimen, even the local ${Ca}_{w}-O_{s}$ bond breaks, and the Ca atom may rapidly reestablish the chemical bond with the adjacent O atom. The chemical bond reconstruction helps to recover the small defects in the elastic area. However, in the saturated sample, the interlayer $O_{w}$ replaced part of the $O_{s}$, forming an unstable ${Ca}_{w}-O_{w}$ bond. Due to the influence of water molecules, the broken ${Ca}_{w}-O_{w}$ connection cannot be rebuilt as easily as the dry CSH gel, as shown in Figure 10. Therefore, the saturated CSH gel has a more brittle structure, which is reflected in the Mode I fracture loaded process on the ECC, as evidenced by the decline in $K_{IC}^{ini}$.

$K_{IC}^{un}$ is SW > SSW > D, while $G_{I}$ is SW > SSW > D. The fiber-matrix interface characteristic plays a major role in the overall performance of fiber-reinforced cement composites [44]. PVA fiber is a hydrophilic material [45], which allows the hydrophilic fiber to fully exert the bonding performance between the matrix and fiber under the lubrication effect of moisture, and the improvement of fiber bonding performance is to promote slippage between the cement base and the fiber substrate. $K_{IC}^{un}$ and $G_{I}$ increase as the saturation rate increases, which is an important factor for hardening [46]. This is distinct from the effect of the saturation rate on the fracture performance of concrete. The $G_{I}$ of concrete decreases as saturation increases [28].

$K_{IC}^{ini}$ is SW > D > SSW at 0 °C, and the water in the pores begins to freeze, significantly improving the mechanical properties of cement-based materials, particularly the weak pore structure [17]. The improving impact is increasingly noticeable as the moisture content increases. However, because the SSW specimen is not completely saturated with water, the amount of icing is small at this time, and only part of the pores is filled. The increase in the SSW specimen is limited, and the overall surface energy of the D specimen is still higher than the SSW specimen. $K_{IC}^{un}$ and $G_{I}$ are both D > SW > SSW. The ductility of D and SSW specimens is greater than SW specimens. This is because PVA fiber is a hydrophilic material, and ice bonds better with hydrophilic materials [47]; if the fiber can be pulled out, the higher the bonding performance, the higher the ductility. However, part of the pore water freezes at this moment. The higher the water content, the more the icing, which leads to excessive bonding performance, pulling out some fibers, and a decrease in ductility. The most significant process for improving $G_{I}$ is fiber pulling [48]. Therefore, as the saturation rate increases, $K_{IC}^{un}$ and $G_{I}$ decrease.

At −20 °C, $K_{IC}^{ini}$ is SW > D > SSW, which means that most of the pore water freezes. The higher the saturation, the more fully the weak part of the matrix is repaired and stronger the fiber-matrix interface bonding force, resulting in a large increase in strength. The energy consumption of the fiber decreased as the number of the fiber pulled segments increased, and the SW and SSW specimens transitioned from ductile to quasi-brittle fracture. $K_{IC}^{un}$ and $G_{I}$ are both D > SSW > SW.

$K_{IC}^{ini}$ and $K_{IC}^{un}$ are both SW > SSW > D at −60 °C. Three levels of saturation test specimens exhibit quasi-brittle failure at this time. In the strain hardening section, the fiber has almost no energy dissipation capacity, and cracks are not constrained by fibers; therefore, they crack quickly. With a higher level of saturation, the “steep” descending segment of the *P-CMOD* curve leads to a rapid decline in $G_{I}$. The matrix has a greater impact on $K_{IC}^{ini}$ and $K_{IC}^{un}$. The enhancement of $K_{IC}^{un}$ by the matrix exceeds the attenuation of by the fiber, indicating an upward trend of $K_{IC}^{un}$.

#### 3.1.2. The Influence of Temperatures on the Fracture Properties of Specimens with Different Saturation

**Figure 12:**
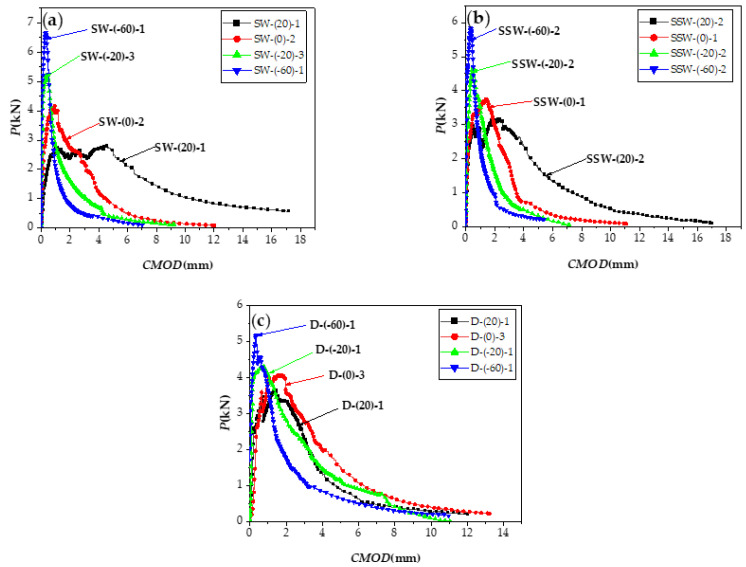
*P-CMOD* curves of (**a**) SW, (**b**) SSW, and (**c**) D series (with temperatures).

**Figure 13:**
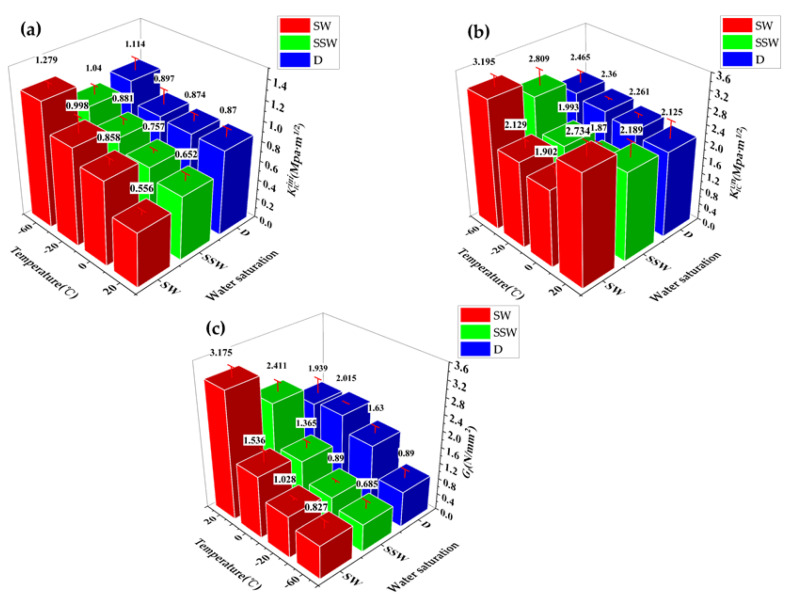
The (**a**) $K_{IC}^{ini}$, (**b**) $K_{IC}^{un}$, and (**c**) $G_{I}$ varies with temperature at three saturation levels.

Figure 12 and Figure 13 shows that, in the saturated state, when temperatures fall, the $K_{IC}^{ini}$ of ECC increases sharply. The following are the four reasons for this. One is that the freezing temperatures of ice vary, depending on pore diameter. The larger pores are frozen first, followed by the smaller pores, and the ice content inside the specimen increases [48]. The ice filling improves the cement-based material and mechanical properties, particularly the weak pore structure. The second reason is that, at low temperature, the shape of ice changes [49], and its compressive and bonding strengths increases as the temperatures decreases [50]. The third point is that ice has a significantly higher bonding strength under tension than it does under shear [47]. The fourth is an improvement in the bonding performance of the fiber-matrix interface, with a focus on the increase in friction in the bonding performance [51]. Therefore, $K_{IC}^{ini}$ is gaining popularity. $K_{IC}^{un}$ decreases at 0 °C, increases at −20 and −60 °C, and $G_{I}$ continues to decrease. The reason for this is the decline in temperatures. Although $K_{IC}^{ini}$ increases, fiber energy consumption decreases. The fiber plays a major role in the early stages. Because the decrease in temperature weakens the fiber’s energy consumption more than the increase in the matrix, the $K_{IC}^{un}$ significantly decreases at 0 °C. There is a temperature point between 0~−20 °C, where the specimen transitions from ductility to quasi-brittleness. The mechanism analysis for −60 and −20 °C is similar. $K_{IC}^{ini}$ and $K_{IC}^{un}$ increase, while $G_{I}$ decreases. After becoming unstable, the fiber cracks and quickly breaks.

In the semi-saturated state, the $K_{IC}^{ini}$, $K_{IC}^{un}$, and $G_{I}$ of the ECC show a similar trend as the saturated state with the decline in temperatures. However, the fracture performance is affected by temperatures and has no obvious changes in water-saturated specimens. The above phenomenon is closely related to its water content.

In the dry state, $K_{IC}^{ini}$ and $K_{IC}^{un}$ increased slowly, while $G_{I}$ increased at 0 °C and decreased at −20 and −60 °C. This could be attributed to the decrease in atom distance at low temperatures, the increase in the attractive force between atoms [28], and the change in fiber performance at low temperatures [51]. For dry specimens, there is a temperature point between −20~−60 °C, which causes the specimens to change from ductile to a quasi-brittle fracture.

### 3.2. The Effect of Freeze–Thaw Cycles on the Fracture Properties When ECC Is Loaded at 20 and −20 °C

**Figure 14:**
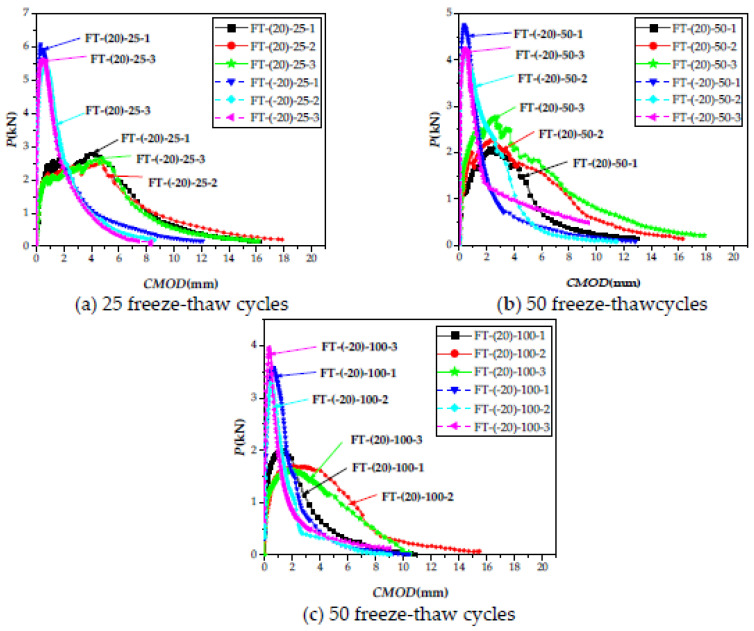
*P-CMOD* curves under 20 and −20 °C, loaded after different freeze–thaw cycles. (**a**) 25 freeze-thaw cycles. (**b**) 50 freeze-thaw cycles. (**c**) 100 freeze-thaw cycles.

Figure 14a–c shows the *P-CMOD* curve loaded at 20 and −20 °C after ECC freeze–thaw for 25, 50, and 100 cycles. Table 6 shows the fracture properties calculation table, with data at 20 °C for zero freeze–thaw cycles, derived from the SW-(20) group and data at −20 °C for zero cycles of freeze–thaw cycles derived from the SW-(−20) group.

**Figure 15:**
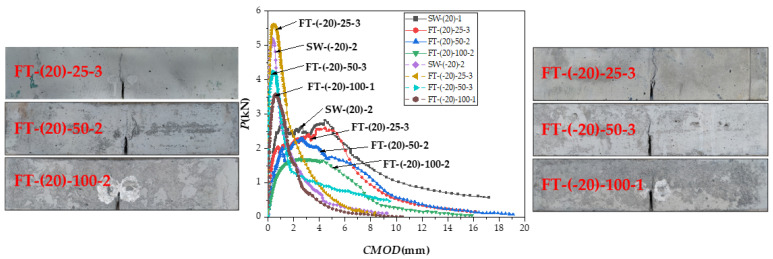
The failure morphology and *P-CMOD* curve after different freeze–thaw cycles.

The failure morphology of the specimen after the freeze–thaw cycles are shown in Figure 15. The number of freeze–thaw cycles increases, yet the inclination angle of the main crack and number of cracks around the prefabricated cut have not obvious regularity.

**Figure 16:**
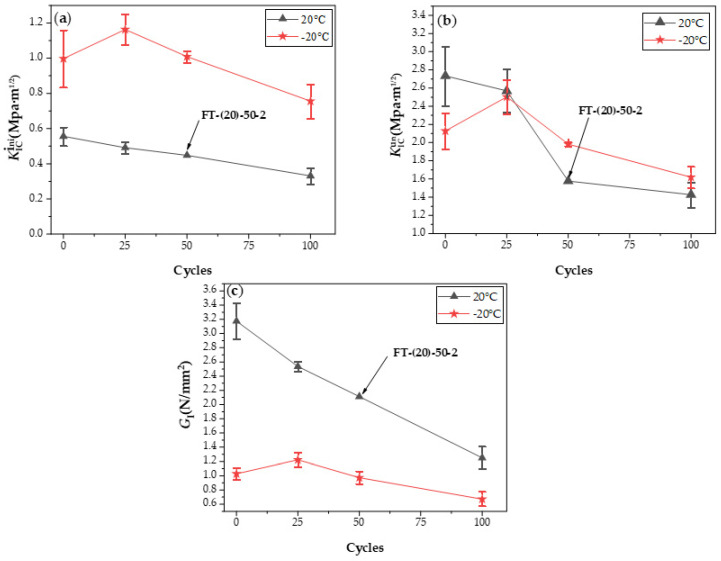
(**a**) $K_{IC}^{ini}$, (**b**) $K_{IC}^{un}$, and (**c**) $G_{I}$ changes after different freeze–thaw cycles at 20 and −20 °C.

As shown in Figure 15 and Figure 16, after 25 freeze–thaw cycles, $K_{IC}^{ini}$, $K_{IC}^{un}$, and $G_{I}$ decrease when loaded at 20 °C, which is related to the degradation of the interface performance between the matrix and fiber-matrix produced by the freeze–thaw cycles [52]. There was little damage to the specimen’s surface, and a few new pores developed; however, the quality and dynamic elastic modulus improved. According to previous studies [53,54], this is due to the micro-ice crystals in the pores, which serve as a cryopump. During the cooling process, water is pumped from the gel pores and micropores to the area of the micro ice crystals, indicating that the specimen is supersaturated during freezing and thawing. The water migrates in the opposite direction throughout the temperatures recovery process. Therefore, it appears to absorb water when there is water outside during the entire freeze–thaw cycle. Therefore, after 25 freeze–thaw cycles, the quality and dynamic elasticity of ECC appear to improve.

The surface mortar begins to fall off after 50 freeze–thaw cycles, the pore structure changes from an elastic to elastoplastic state, there is irreversible residual strain accumulation [55], and it produces excessive residual plastic strain [4]. In the case of tensile strength, when the water swelling stress exceeds the resistance of the ECC matrix, the internal water swelling stress is released through micro cracks. The freeze–thaw cycles damage the matrix and destroy the fiber-matrix interface [4], shorten the peak displacement when the fiber slips out of the matrix [56], and cause chemical adhesion and reduced friction at the same time. The fiber is still pulled out, not pulled off, and the strain hardening effect still exists.$K_{IC}^{ini}$, $K_{IC}^{un}$, and $G_{I}$ decline, but still have a certain level of ductility. The surface cement paste drastically dropped after 100 freeze–thaw cycles, fibers leaked out, and fracture properties deteriorated once more.

ECC faces two types of coupling damage once the freeze–thaw cycles are loaded at −20 °C: low temperatures and freeze–thaw. Low temperatures and freeze–thaw damage are assumed to be linearly superimposed (LS). The result of linear superposition of the theoretical value at −20 °C after the freeze–thaw cycles is recorded as LSFT-(−20)-(N). *N* represents the number of cycles, the calculation formula of $G_{I(LSFT-(-20)-\left(N)\right)}$ is shown in Formula (14), and the calculated value is shown in Table 7.

(14) $$\begin{array}{c} G_{I(LSFT-(--20)-(N))}=(1-\frac{G_{I(SW-(20))}-G_{I(SW-(-20))}}{G_{I(SW-(20))}})\cdot G_{I(FT-(20)-(N))} \end{array}$$

where $G_{I(LSFT-(-20)-(N))}$ is the −20 °C low temperature and freeze–thaw cycles are superimposed linearly nominal fracture energy, $G_{I(SW-(20))}$ is the nominal fracture energy of the SW-(20) group, and $G_{I(SW-(-20))}$ is the nominal fracture energy of the SW-(−20) group.

**Figure 17:**
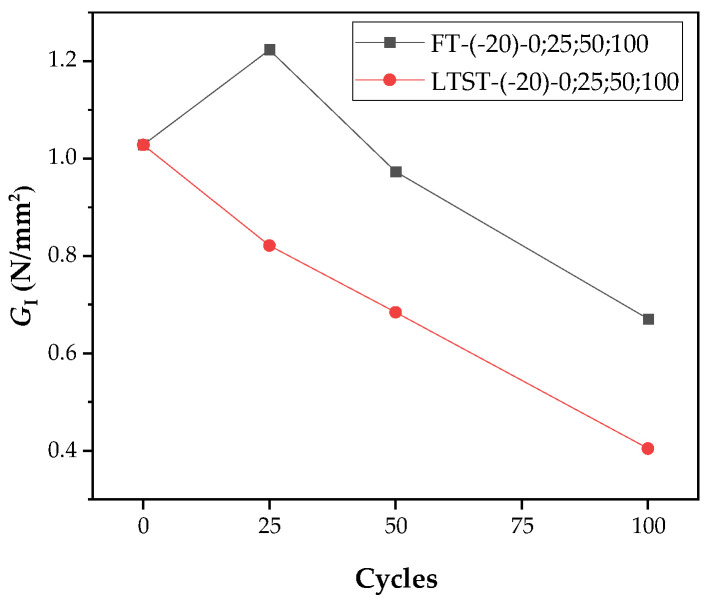
Comparison of $G_{I}$ between FT and LSFT after freeze–thaw cycles.

Contrast with test values at −20 °C after freeze–thaw cycles, as shown in Figure 17, when the number of freeze–thaw cycles are the same, the value of $G_{I(FT-(20)-(N))}$ is higher than $G_{I(LSFT-(-20)-(N))}$.

This may be due to the freeze–thaw cycles, which causes more water to enter the matrix and make greater contact with the fiber. The fiber is pulled off like the SW-(−20) group of specimens, when loaded at −20 °C. At −20 °C, however, more ice is formed, and the contact area between the fiber and ice increases, which enhances bonding performance, particularly friction. Simultaneously, too much ice further repairs the damaged matrix, and the increase in the energy dissipation capacity of the matrix is greater than the attenuation of the energy dissipation capacity of the fiber. Therefore, the two damages are not linearly superimposed.

**Figure 18:**
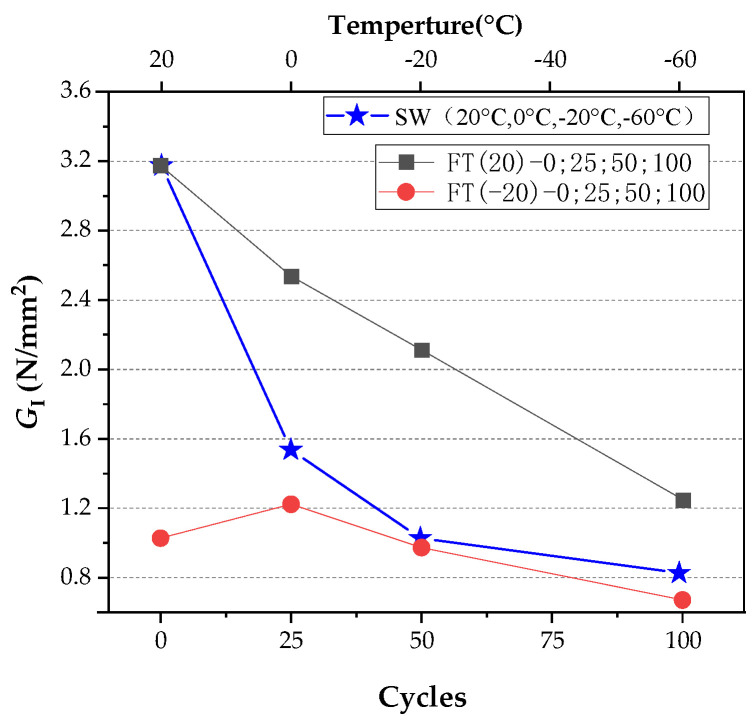
Comparison of $G_{I}$ of SW series at four target temperatures and $G_{I}$ of test group after different freeze–thaw cycles at 20 and −20 °C.

As shown in Figure 18, at low temperatures, the SW group $G_{I}$ decreases by 67.6% at 20 to −20 °C, and only 19.5% at −20 to −60 °C; however, the $G_{I}$, loaded at 20 °C, continues to linearly decrease, after the freeze–thaw cycles. After more than 25 cycles, the $G_{I}$ of FT-(20)-100 group is close to that of SW-(−20) group. As the number of freeze–thaw cycles increases, the $G_{I}$ damage to ECC caused by freeze–thaw will becomes more severe, compared to the damage at low temperatures.

## 4. Conclusions

In the present study, the prefabricated notch three-point bending beams were used to study the fracture properties of ECC Mode I under low-temperature conditions with different target temperatures, different water saturation and after freeze–thaw cycles, and loaded at 20 and −20 °C. The main conclusions are as follows:

(1) Before the temperature drops to the internal pore water of ECC before freezing, the increase of internal moisture content has a negative impact on the strength of ECC matrix, while it is conducive to the slip hardening of the fibers in the matrix. Therefore, the ECC is easy to crack and has better energy dissipation capacity. For example, when loaded at 20 °C, the fracture parameters are affected by moisture, and the specific performance decreased and showed the opposite trends.
(2) When the temperature drops to the freezing temperature of pore water inside ECC, the lower the temperature and more ice content, the ECC will be less prone to cracking. The ECC with higher water saturation had more ice content inside, leading to the increase of fiber-matrix interface adhesion. On the other hand, the attractive force between the ice strength and atoms increases as the temperature decreases, which was manifested by the continuous increase at 0, −20, and −60 °C, and the higher the moisture content, the more increases.
(3) With the continuous decrease of ECC with temperature, the failure mode changes from ductile to a quasi-brittle fracture. The phenomenon was also affected by the moisture content; that is, the lower the moisture content, the lower the temperature range from ductile to a quasi-brittle fracture. For example, it can be seen from the P-CMOD curve that there is a temperature point at 0 °C~−20 °C for saturated and semi-saturated specimens, which makes the ECC change from ductile fracture to quasi-brittle fracture, while the temperature point of the dry specimens exists at −20 °C~−60 °C. This phenomenon is closely related to the continuous enhancement of the adhesion between the PVA fiber and matrix, with the decrease of temperature. Excessive adhesion leads to more fibers being pulled off during the fracture process and is unable to exert energy dissipation capacity.
(4) The influence of moisture on ECC and concrete energy dissipation capacity showed the opposite trend. When the pore water is not frozen, the moisture raised the ECC, while it dropped off the concrete. However, after the pore water is frozen, the ECC dropped and the concrete raised.
(5) The linear superposition assumption did not apply to the calculation of the damage coupled with ECC freeze–thaw and low temperature. The theoretical value of the linear superposition was lower than the experimental value, which may be related to the supersaturation effect and different damage mechanisms of freeze–thaw and low temperature to ECC under saturated state. Freezing and thawing will deteriorate the matrix strength and fiber matrix interface bonding properties, while the fiber can still be pulled out, and the strain hardening segment still exists. At low temperatures, the strength of matrix was improved, bonding properties of fiber-matrix interface were enhanced, fiber was pulled off, and strain hardening segment gradually disappeared.

## Figures and Tables

**Table 1:** Composition content of fly ash and silica fume.

Chemical Composition (%)	SiO_2_	Al_2_O_3_	Fe_2_O_3_	CaO	MgO	SO_3_	K_2_O	Na_2_O	TiO_2_	H_2_O
Fly ash (%)	62	33	2	0.2	0.9	0.9	1			
Silica fume (%)	94.03	0.31	0.46	0.02	0.83		2.08	1.31	0.04	0.92

**Table 2:** Parameters of PVA fiber. Reproduced with permission from [Shuling Gao], [International Journal of Solids and Structures], [ELSEVIER], [2022].

Diameter (mm)	Length (mm)	Length Diameter ratio	Extensibili-ty (%)	Tensile Strength (MPa)	Modulus of Elasticity (GPa)	Density (kg/m^3^)
0.04	12	300	6	1600	40	1300

**Table 3:** Mixture proportions (kg/m^3^).

Material Name	Cement	Fly Ash	Silica Fume	Quartz Sand	Water	Acid Superplasticizer	PVA Fiber
ECC	0.20	0.78	0.02	0.25	0.30	0.10%	2%

Note: Volume content is used for PVA fibers, and weight ratio is used for other constituent materials. The dosage of acid superplasticizer is expressed as a percentage of the weight of the binding material.

**Table 4:** Serial number of specimens.

Number	Cycles	Saturation	Temperatures (°C)	Number of Test Specimens
SW-(20)	0	Saturated water	20	3
SW-(0)	0	Saturated water	0	3
SW-(−20)	0	Saturated water	−20	3
SW-(−60)	0	Saturated water	−60	3
SSW-(20)	0	Semi saturated water	20	3
SSW-(0)	0	Semi saturated water	0	3
SSW-(−20)	0	Semi saturated water	−20	3
SSW-(−60)	0	Semi saturated water	−60	3
D-(20)	0	Dry	20	3
D-(0)	0	Dry	0	3
D-(−20)	0	Dry	−20	3
D-(−60)	0	Dry	−60	3
FT-(20)-25	25	Saturated water	20	3
FT-(−20)-25	25	Saturated water	−20	3
FT-(20)-50	50	Saturated water	20	3
FT-(−20)-50	50	Saturated water	−20	3
FT-(20)-100	100	Saturated water	20	3
FT-(−20)-100	100	Saturated water	−20	3

Note: SW-T; SSW-T; D-T; SW stands saturated water; SSW stands semi saturated water; D stands dry; T stands temperature; FT-T-N, FT stands freeze–thaw; N stands number.

**Table 5:** ECC fracture properties calculation table, with three levels of saturation in four target temperatures.

Satura-tion Levels	Number	Temperatures (°C)	$P_{\mathrm{ini}}$ (kN)	$P_{\max}$ (kN)	$K_{\mathrm{IC}}^{\mathrm{ini}}\;(\mathrm{MPa}\cdot^{1/2})$	$K_{\mathrm{IC}}^{\mathrm{un}}\;(\mathrm{MPa}\cdot^{1/2})$	$G_{I}\;(N/{\mathrm{mm}}^{2})$
SW	SW-(20)-1	20	1.463	2.828	0.496	2.482	3.394
	SW-(20)-2	20	1.831	2.615	0.592	2.617	2.902
	SW-(20)-3	20	1.787	3.24	0.581	3.103	3.229
	Average value		1.694	2.894	0.556	2.734	3.175
	SW-(0)-1	0	2.628	4.059	0.801	1.736	1.252
	SW-(0)-2	0	2.777	4.201	0.84	1.906	1.515
	SW-(0)-3	0	3.123	4.506	0.931	2.064	1.842
	Average value		2.843	4.255	0.858	1.902	1.537
	SW-(−20)-1	−20	3.949	5.543	1.148	2.345	1.002
	SW-(−20)-2	−20	2.716	4.875	0.825	1.959	1.116
	SW-(−20)-3	−20	3.464	5.218	1.021	2.081	0.964
	Average value		3.376	5.212	0.998	2.129	1.028
	SW-(−60)-1	−60	4.551	6.663	1.306	3.126	0.749
	SW-(−60)-2	−60	4.585	6.398	1.315	3.481	1.052
	SW-(−60)-3	−60	4.204	7.099	1.215	2.978	0.679
	Average value		4.447	6.720	1.279	3.195	0.827
SSW	SSW-(20)-1	20	1.893	2.95	0.608	2.321	2.261
	SSW-(20)-2	20	2.389	3.149	0.739	2.486	2.266
	SSW-(20)-3	20	1.891	3.254	0.608	1.762	2.708
	Average value		2.058	3.118	0.652	2.189	2.411
	SSW-(0)-1	0	2.608	3.771	0.796	2.115	1.236
	SSW-(0)-2	0	2.301	3.919	0.716	1.58	1.291
	SSW-(0)-3	0	2.464	3.56	0.758	1.913	1.568
	Average value		2.458	3.750	0.757	1.87	1.365
	SSW-(−20)-1	−20	3.125	4.444	0.932	1.934	0.955
	SSW-(−20)-2	−20	3.024	4.683	0.905	2.009	0.887
	SSW-(−20)-3	−20	2.645	5.241	0.806	2.036	0.829
	Average value		2.931	4.789	0.881	1.993	0.89
	SSW-(−60)-1	−60	3.714	6.732	1.087	3.253	0.901
	SSW-(−60)-2	−60	3.763	5.857	1.099	2.52	0.659
	SSW-(−60)-3	−60	3.135	5.656	0.935	2.653	0.495
	Average value		3.537	6.082	1.04	2.809	0.685
D	D-(20)-1	20	2.517	3.649	0.772	2.067	1.559
	D-(20)-1	20	2.976	3.613	0.893	1.726	2.294
	D-(20)-1	20	3.174	4.346	0.945	2.582	1.964
	Average value		2.889	3.869	0.87	2.125	1.939
	D-(0)-1	0	3.182	4.453	0.947	2.277	2.024
	D-(0)-2	0	2.921	3.908	0.878	2.351	1.993
	D-(0)-3	0	2.612	4.069	0.797	2.156	2.029
	Average value		2.905	4.143	0.874	2.261	2.015
	D-(−20)-1	−20	2.779	4.342	0.841	2.395	1.811
	D-(−20)-2	−20	2.543	3.649	0.779	2.31	1.627
	D-(−20)-3	−20	3.655	4.464	1.071	2.375	1.454
	Average value		2.992	4.151	0.897	2.36	1.63
	D-(−60)-1	−60	3.449	5.175	1.017	2.247	1.061
	D-(−60)-2	−60	3.671	4.78	1.075	2.362	0.649
	D-(−60)-3	−60	4.34	5.634	1.251	2.784	0.962
	Average value		3.820	5.196	1.114	2.465	0.89

Note: $P_{ini}$, measured by strain gauges at 20 °C, is shown in Figure 9a. Low temperature (0, −20, and −60 °C) crack initiation forces, measured by *P-CMOD*, *P-CTOD* turning point.

**Table 6:** ECC fracture properties calculation table after different freeze–thaw cycles.

Cycles	Number	Temperatures (°C)	$P_{\mathrm{ini}}$ (kN)	$P_{\max}\;(\mathrm{kN})$	$K_{\mathrm{IC}}^{\mathrm{ini}}\;(\mathrm{MPa}\cdot^{1/2})$	$K_{\mathrm{IC}}^{\mathrm{un}}\;(\mathrm{MPa}\cdot^{1/2})$	$G_{I}\;(N/{\mathrm{mm}}^{2})$
25	FT-(20)-25-1	20	1.591	2.8	0.529	2.34	2.581
	FT-(20)-25-2	20	1.395	2.582	0.478	2.552	2.573
	FT-(20)-25-3	20	1.35	2.597	0.466	2.815	2.456
	Average value		1.445	2.660	0.491	2.569	2.537
	FT-(−20)-25-1	−20	4.334	6.083	1.249	2.353	1.158
	FT-(−20)-25-2	−20	3.674	5.465	1.076	2.71	1.171
	FT-(−20)-25-3	−20	4.014	5.624	1.165	2.447	1.342
	Average value		4.007	5.724	1.163	2.503	1.223
50	FT-(20)-50-1	20	1.362	2.125	0.469	1.707	1.835
	FT-(20)-50-2	20	1.282	2.308	0.448	1.577	2.113
	FT-(20)-50-3	20	1.199	2.77	0.426	1.794	2.311
	Average value		1.281	2.401	0.448	1.692	2.086
	FT-(−20)-50-1	−20	3.512	4.771	1.033	2.007	0.877
	FT-(−20)-50-2	−20	3.47	4.179	1.001	1.983	0.973
	FT-(−20)-50-3	−20	3.274	4.258	0.971	1.965	1.05
	Average value		3.419	4.403	1.009	1.984	0.973
100	FT-(20)-100-1	20	0.895	2.001	0.347	1.288	1.114
	FT-(20)-100-2	20	0.631	1.728	0.277	1.426	1.419
	FT-(20)-100-3	20	0.982	1.621	0.369	1.566	1.217
	Average value		0.836	1.783	0.331	1.426	1.249
	FT-(−20)-100-1	−20	2.213	3.595	0.693	1.71	0.787
	FT-(−20)-100-2	−20	2.278	3.291	0.709	1.487	0.607
	FT-(−20)-100-3	−20	2.873	3.98	0.866	1.662	0.62
	Average value		2.455	3.622	0.758	1.620	0.671

Note: The initiation load was measured by strain gauges at 20 °C, with 25 freeze–thaw cycles. The outer surface of the specimen is severely damaged in 50 and 100 cycles. *P-CMOD* and *P-CTOD* are used to determine the initiation load. At −20 °C, the crack initiation load is determined according to *P-CMOD* and *P-CTOD*. The test methods are shown in Figure 9a–c.

**Table 7:** Linear superposition fracture energy calculation.

Cycles	0	25	50	100
$G_{I(LSFT-(-20)-(N))}$ (N/mm^2^)	1.028	0.821	0.684	0.405

## Data Availability

The data used to support the findings of this study are available from the corresponding author upon request.

## Abbreviations

a	initial crack length
$a_{c}$	effective elastic crack length
$A_{tig}$	area of the broken ligament
b	thickness of specimen
c	height of specimen
$c_{i}$	initial compliance coefficient
CMOD	crack mouth opening displacement
${CMOD}_{0}$	*CMOD* value when the bearing capacity drops to 20% of the peak load
CTOD	crack tip opening displacement
D	dry
E	elastic modulus
ECC	engineered cementitious composites
$f_{0i}$	initial value of the transverse fundamental frequency of specimen
$f_{ni}$	transverse fundamental frequency of the specimen after *n* freeze–thaw cycles
FT	freeze–thaw
$G_{I}$	nominal fracture energy
$G_{I(SW-(20))}$	nominal fracture energy of the SW-(20) group
$G_{I(SW-(-20))}$	nominal fracture energy of the SW-(−20) group
$h_{0}$	thickness of the blade used to fix the clip
h	height of specimen
$K_{IC}^{ini}$	initial fracture toughness
$K_{IC}^{un}$	nominal unstable fracture toughness
L	length of specimen
LS	linearly superimposed
$m_{1}$	mass of specimen
$m_{2}$	weight of the loading head not fixed on the testing machine
$M_{0}$	initial mass of specimen
$M_{1}$	mass of the specimen after soaked in water
N	number of cycles
P	applied load
$P_{ini}$	initial cracking load
$P_{max}$	peak load
$P_{i}$	dynamic elastic modulus of the specimen after freeze–thaw cycles
S	net span of the test specimen
*SW*	saturated water
*SSW*	semi saturated water
T	temperature
*W_0_*	mass of the specimen after n freeze–thaw cycles
*W_n_*	mass loss rate of the specimen after n freeze–thaw cycles
δ	midspan deflection
α	relative crack extension length and $\alpha =(a+h_{0})/\left(h+h_{0}\right)$
$\alpha_{c}$	relative crack extension length and $\alpha_{c}=(a_{c}+h_{0})/\left(h+h_{0}\right)$
